# Whole-organ and segmental stiffness measured with liver magnetic resonance elastography in healthy adults: significance of the region of interest

**DOI:** 10.1007/s00261-014-0278-7

**Published:** 2014-10-21

**Authors:** Grażyna Rusak, Elżbieta Zawada, Adam Lemanowicz, Zbigniew Serafin

**Affiliations:** Department of Radiology and Diagnostic Imaging, Nicolaus Copernicus University, Collegium Medicum, ul. Skłodowskiej-Curie 9, 85-094 Bydgoszcz, Poland

**Keywords:** Liver, Stiffness, MR elastography, Variability

## Abstract

**Purpose:**

MR elastography (MRE) is a recent non-invasive technique that provides in vivo data on the viscoelasticity of the liver. Since the method is not well established, several different protocols were proposed that differ in results. The aim of the study was to analyze the variability of stiffness measurements in different regions of the liver.

**Methods:**

Twenty healthy adults aged 24–45 years were recruited. The examination was performed using a mechanical excitation of 64 Hz. MRE images were fused with axial T2WI breath-hold images (thickness 10 mm, spacing 10 mm). Stiffness was measured as a mean value of each cross section of the whole liver, on a single largest cross section, in the right lobe, and in ROIs (50 pix.) placed in the center of the left lobe, segments 5/6, 7, 8, and the parahilar region.

**Results:**

Whole-liver stiffness ranged from 1.56 to 2.75 kPa. Mean segmental stiffness differed significantly between the tested regions (range from 1.55 ± 0.28 to 2.37 ± 0.32 kPa; *P* < 0.0001, ANOVA). Within-method variability of measurements ranged from 14 % for whole liver and segment 8–26 % for segment 7. Within-subject variability ranged from 13 to 31 %. Results of measurement within segment 8 were closest to the whole-liver method (ICC, 0.84).

**Conclusions:**

Stiffness of the liver presented significant variability depending on the region of measurement. The most reproducible method is averaging of cross sections of the whole liver. There was significant variability between stiffness in subjects considered healthy, which requires further investigation.

Liver biopsy is a reference standard for detection and staging of the liver fibrosis nowadays. However, this procedure is not always well tolerated by patients; it can result in complications and is a subject to sampling error and interpretation errors [1]. Since liver fibrosis results in a loss of elasticity or viscoelasticity, several methods have been introduced that offer an indirect estimation of these parameters. They include transient elastography (TE), acoustic radiation force impulse imaging (ARFI), and magnetic resonance elastography (MRE) [1–4].

The most widely used method for the liver stiffness assessment is ultrasound (US) elastography, mainly because of its low cost, short scanning time, and wide availability. However, this method is strongly operator dependent, and there is a wide range of different scanning techniques which are likely not equivalent [5, 6]. As a result, diagnostic reference values are system specific and cannot be directly compared across different systems. Currently, TE is considered the best-studied US technique for the liver stiffness assessment [6].

Another imaging option for non-invasive evaluation of the liver fibrosis is MRE, which is thought to overcome some of US shortcomings and is capable to determine viscoelastic properties of the tissue [7]. Viscoelasticity, which is indirectly measured as stiffness, is known as a strong predictor of liver fibrosis presenting a very high negative predictive value of 97 % [8]. Since the method is not well established, several different protocols have been proposed in the literature with various approaches concerning the optimal place of stiffness measurement [8–12]. In a fundamental paper by Yin et al., shear stiffness of the liver was measured in a region of interest (ROI) that included an entire axial cross section of the liver, excluding major blood vessels that were defined as larger than 6 pixels [8]. On the other hand, Venkatesh et al. placed small ROIs (of at least 100 mm^2^) in segment 8 avoiding the liver edge, fissures and vessels over 3 mm large, and focal lesions. [9]. The segment 8 was chosen to limit stiffness measurement to the most common location of the liver biopsy. By contrast, Bothe et al. measured stiffness on a single ROI that roughly covered the right liver lobe [10]. Finally, Ichikowa et al. put only one ROI, which measured at least 1.5 cm^2^ and was located “in the areas with no vessels and in a distance from liver edge” [11].

Moreover, several authors reported that MRE presents significant a variability of results. Even when using a regular system of ROI placement, examinations of the same healthy subjects present variability of 12–27 % [12, 13]. Since reproducibility determines the magnitude of changes that may be considered clinically significant, the aim of the study was to analyze the variability of stiffness measurements between different regions of the liver.

## Materials and Methods

The study was approved by a local bioethical committee. Sample size was calculated according to Kelley K. [14], assuming for 90 % Confidence Interval (CI) for the calculated coefficient of variation (CV) of the measured liver stiffness, a 15 % population CV, a 0.8 desired degree of assurance for achieving a CI no wider than 15 %, and a desired full CI width of 10 %.

Inclusion criteria were informed consent for the participation and a normal liver image on ultrasound. Sonographic examination was performed by a radiologist with 13 years of experience in abdominal imaging using Toshiba Xario unit with a convex probe (2–5 MHz).

Exclusion criteria were as follows: serum level of alanine transaminase over 40 IU/L, liver pathologies in anamnesis, any known risk factors of liver disease, any previous hospital stay, any previous surgical procedures, a regular diet for at least 6 months, no alcohol abuse (less than 20 g of pure alcohol per day), overweight or obesity, and common contraindications to MRI (uncontrolled claustrophobia, an implanted pacemaker/ICD, any ferromagnetic foreign bodies, etc.).

Twenty-four subjects were screened using ultrasound for the potential participation in the study. Four of them were excluded due to detection of hemangiomas (2 subjects) and steatosis (2). Thus, twenty healthy adults aged 24–45 years (mean age 39.1 years) were recruited for this prospective pilot study. Examinations were performed in a supine position after a 4-hour fasting. A 1.5 T scanner (Optima 450w, GE Healthcare, Waukesha, WI) with a 16-channel abdominal phased array coil and an MR elastography system (MR-Touch, GE Healthcare) was used. Before MRE, standard axial T2WI imaging was performed to rule out any morphological pathologies of the liver. None of subjects were excluded at this stage.

A passive driver of 19 cm in diameter was placed on the lower chest wall and the upper abdomen, on the right side, with the driver’s central point located at the level of the xiphisternum—over the right lobe of the liver. Acoustic vibrations of 60 Hz generated and transmitted by the system produced shear waves in the epigastrium and the liver. The output power was manually adjusted (30–50 % of the peak capacity of the driver) to ensure sufficient penetration of shear waves within the liver and was within the limits of safety, according to the European Union directive on occupational exposure to whole-body and extremity vibrations.

The propagation of the shear waves within the liver was imaged with a two-dimensional GRE MR elastography sequence (TE 50 ms, TR 19.2 ms, flip angle 30°, BW 31.25 kHz, matrix 64 × 64, slice thickness 10 mm, spacing 10 mm) on a breath hold at end-expiration. MRE images (stiffness maps) were fused with axial T2WI breath-hold images of the same slice thickness. No intravenous contrast was given. Images were evaluated by two radiologists with 3 years of experience in liver MR imaging, and all discrepancies were solved by a consensus.

**Fig. 1:**
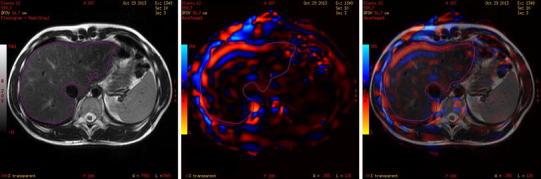
Single cross section of the liver. Whole-liver stiffness was calculated as a mean of such ROIs drawn on all axial slices of the liver. The ROI is presented on T2-weighted image (*left*), elastogram (*middle*), and fused image of T2WI and elastogram (*right*).

**Fig. 2:**
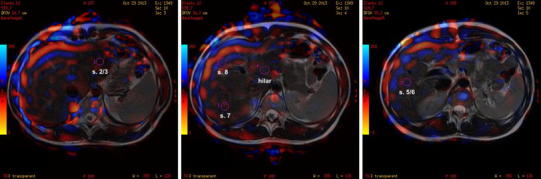
Measurement of segmental stiffness on fused image of T2WI and elastograms.

The stiffness was measured on 8 axial slices. The middle (5th) slice was placed at the bifurcation of the portal vein and served as a reference slice in each patient. Whole-liver stiffness was measured using consecutive ROIs, which covered slices of the whole liver, excluding the inferior vena cava and the gallbladder. Therefore, stiffness was an average of 8 cross sections of the liver (Fig. 1). Cross-sectional stiffness measured according to the method by Lee et al. [15]. It was calculated in a ROI that was placed on a single largest cross section of the liver and was excluding great hepatic vessels; it accounted for approximately 70 % of the entire cross section of the liver. Right lobe stiffness was measured on the largest single cross section, excluding hepatic great vessels. Additionally, segmental stiffness was measured in 5 small ROIs (50 pix.—approx. 150 mm^2^) that were placed in the center of the left lobe (segments 2/3), segments 5/6, 7, 8, and the parahilar region (Fig. 2). ROIs were placed centrally within the liver parenchyma avoiding large hepatic vessels.

The results were expressed as mean stiffness (kPa) ± standard deviation. Normality of data was tested with Shapiro–Wilk test. The relation between values was determined using Pearson correlation coefficient (*r*). The significance of differences in the stiffness between the tested regions was tested with ANOVA and with *t* test for a direct comparison between regions. Relative variability of stiffness measurement using a particular method (within-method variability) was calculated separately for each method as a CV that was a ratio between the mean value across examined subjects and the standard deviation of the mean. Within-subject relative variability of measurements using small segmental ROIs (left lobe, hilum, segment 5/6, segment 7, and segment 8) was calculated as CV, i.e., it was a ratio between the mean value of stiffness across segments in a single subject and the standard deviation of the mean. Agreement between results of whole-liver measurement and those of particular analyzed regions was tested using intraclass correlation coefficient (ICC, two-way model, absolute agreement for average measures). A P-value of <0.05 was considered significant. Statistical analyses were performed using Statistica 10 (StatSoft Inc., Tulsa, OK) and MedCalc Statistical Software version 13.3 (MedCalc Software bvba, Ostend, Belgium)

## Results

**Fig. 3:**
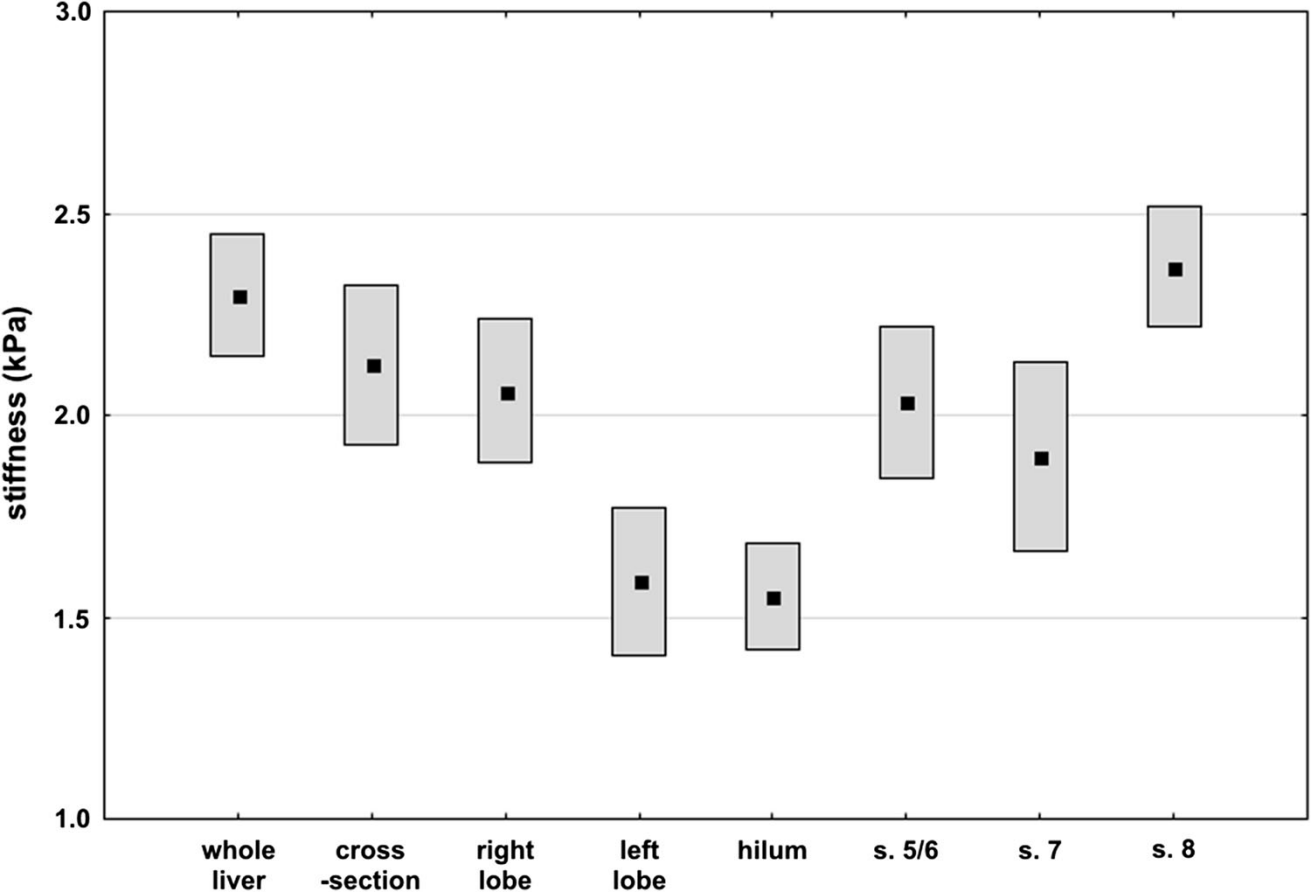
Results of measurements of whole-liver and regional stiffness. The graph presents mean values and their 95 % confidence intervals.

All examinations were performed without complications, and none of them were excluded due to insufficient image quality. Liver stiffness values ranged from 1.10 to 3.08 kPa. Whole-liver stiffness ranged from 1.56 to 2.75 kPa. Mean segmental stiffness differed significantly between the tested regions (range from 1.55 ± 0.28 to 2.37 ± 0.32 kPa; *P* < 0.0001, ANOVA)—Fig. 3, Table 1.

**Table 1:** Mean regional stiffness, within-method variability, and agreement with whole-liver stiffness

Method	Mean Stiffness (± SD)	Within-method variability (%)	ICC (95 % CI) versus cross sections
Whole liver	2.30 (0.32)	14	–
Cross section	2.13 (0.42)	20	0.72 (0.51 to 0.89)
Right lobe	2.06 (0.38)	18	0.69 (0.01 to 0.091)
Left lobe	1.59 (0.39)	25	0.26 (−0.19 to 0.64)
Hilum	1.55 (0.28)	18	0.14 (−0.14 to 0.49)
Segment 5/6	2.03 (0.40)	20	0.68 (0.02 to 0.89)
Segment 7	1.90 (0.50)	26	0.56 (−0.17 to 0.84)
Segment 8	2.37 (0.32)	14	0.84 (0.59 to 0.93)

Considering small ROIs measurements, the stiffness of the left lobe did not correlate with that of the other tested regions. The stiffness of the hilum weakly correlated to that of segments 5/6, 7, and 8 (r, 0.46, 0.55, and 0.52, respectively; *P* < 0.05). The stiffness of segment 5/6 significantly positively correlated to that of segments 7, 8, and the parahilar region (*r*, 0.68, 0.66, and 0.46, respectively; *P* < 0.05). The stiffness of segment 7 significantly positively correlated to that of segments 5/6, 8, and the parahilar region (*r*, 0.68, 0.71, and 0.55, respectively; *P* < 0.05). The stiffness of segment 8 significantly positively correlated to that of segments 5/6, 7, and the parahilar region (*r*, 0.66, 0.71, and 0.52, respectively; *P* < 0.05). Multiple regression analysis revealed that the whole-liver stiffness independently moderately correlated to that of the left lobe and segment 8 (partial r, 0.59, and 0.62, respectively; *P* < 0.02).

The mean whole-liver stiffness was significantly higher than that of the right lobe the left lobe, hilum, segments 5/6, and 7 (*P* < 0.04)—Fig. 3.

**Fig. 4:**
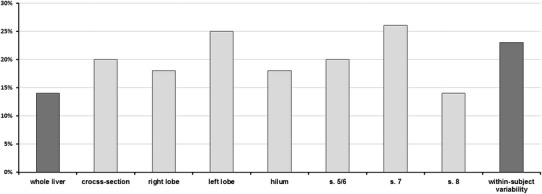
Comparison of within-method and within-subject relative variability of measurements.

Within-method variability of measurements ranged from 14 % for whole liver and segment 8–26 % for segment 7—Fig. 4. Within-subject variability of stiffness measurements ranged from 13 to 31 %, depending on the subject. Results of measurement within segment 8 were closest to the whole-liver method (ICC, 0.84) with other territories presenting at least moderate agreement (Table 1).

## Discussion

We found that the liver segments differ significantly regarding their stiffness as measured with MRE and regarding the inter-subject measurement variability. We also sought that the stiffness averaged between all liver cross sections seems to present the lowest variability and that there is significant variability between stiffness in subjects considered healthy. Moreover, to our knowledge, this is the first report on liver MRE stiffness values among healthy volunteers from Central Europe.

In our small population, including healthy Polish volunteers, the mean whole-liver stiffness was 2.30 kPa. As compared to the published data from Asian populations, these values seem relatively high, e.g., Lee et al. found mean stiffness of 2.12 kPa in their group of Koreans [15], and Venkatesh et al. obtained 2.09 kPa in citizens of Singapore [16]. On the other hand, Hines et al., who studied 20 healthy Americans, reported the mean stiffness at the level of 2.44 kPa [12]. Although based on a limited number of subjects, these data suggest that liver stiffness presents inter-racial variation as well. This variation may depend on genetic differences and environmental factors, including lifestyle, eating habits, and alcohol intake.

Differences between stiffness values using different locations and sizes of ROI have not been extensively investigated yet. Lee et al. performed measurements on a central liver section slice including hepatic hilum and most of the liver parenchyma with 6–10 mm slice thickness [15]. They compared stiffness values measured with (i) a 2-cm ROI placed in the most homogenous part of the slice (probably segment VI/VII), (ii) four averaged 1 cm ROIs located in segments 2/3, 4, 6/7, and 5/8, and (iii) a large ROI that covered approximately 70 % of the cross-sectional area that included the greatest part of the liver parenchyma, excluding hepatic hilar vessels. In contrast, we decided to increase the resolution of the stiffness estimation placing ROIs in a more anatomical way that would reflect a practical surgical approach to the liver. On the other hand, our cross-sectional ROIs did not exclude hepatic vessels as we assumed that subjective determination of vessel margins would introduce a significant operator bias. Thus, the proposed method of stiffness measurement could be considered as a surrogate of 3D liver elastography. In general, the results of the current study confirm general conclusion of Lee et al. that large cross-sectional ROI presents significantly better reproducibility than small segment-specific ROIs.

We found that stiffness differs significantly between the liver segments in particular healthy individuals, which had not been reported previously. Apparently, these differences may be related to the volume of the segment, and its location as structures adjacent to the main liver vessels and rib arch may present altered mechanical properties. Moreover, we found that the range of stiffness values (expressed as variability between patients) that was seen in our study group varied significantly between tested segments. The observation, although not very unexpected, provides scientific evidence for the necessity of precise selection of ROIs for liver stiffness measurements.

Our results indicate that the lowest measurement variability was related to the method, in which stiffness was averaged between all cross sections of the liver. This approach may be considered for use to determine the whole-liver stiffness or as a surrogate of 3D stiffness. An approach to the real liver 3D elastography has recently been presented by Guo et al. [17]. They measured stiffness without excluding major vessels, which was similar to our method, but used a 2.5 mm^3^ voxel resolution and a frequency ranging from 30 to 60 Hz. The mean stiffness in that study was 1.27 kPa with SD of 0.17 kPa, which gives a variability (not reported by authors) of 13.4 %. Although the variability is close to ours, the mean stiffness differs significantly which may be a result of multi-frequency mechanical excitation. In fact, 3D-elastography seems to be the most efficient way to determine the liver stiffness as it enables introduction of software-based automatic segmentation and a more profound investigation of the nature of liver fibrosis. On the other hand, when measured in the same locations, liver stiffness seems to present a high inter-examination reproducibility. In a study by Shi et al., the mean overall ICC for studies repeated in 7 ± 2 days was 0.96 and for studies repeated in 195 ± 15 days was 0.87 [18].

Apart from the selection of the region of measurement also scanning settings may influence results of stiffness assessment in MRE. However, this relation was investigated in few studies. Shinagawa et al. tried to optimize scanning settings of MRE at 3 T in a group of 10 healthy volunteers using magnetic encoding gradient frequencies from 60 to 120 Hz, external driver frequency of 50–70 Hz, slice thickness of 8 mm and 10 mm, and driver amplitudes of 50 and 70 % [19]. The measure of scan quality was the repeatability of the liver stiffness measurement. As a result of the study, the Authors recommend parameters as follows: external acoustic vibration frequency and amplitude 60 Hz and 50 %, respectively, MEG frequency 80 Hz, and slice thickness 8–10 mm. Another approach to MRE is a three-dimensional multifrequency elastography, which offers several new parameters that give deeper insight into the mechanical constitution and the architectural organization of tissues [20]. However, Asbach et al. reported that the diagnostic accuracy of the metrics calculated from multifrequency MRE did not exceed the diagnostic performance obtained with the best single-frequency data or results from the current literature [21]. They also found that metrics obtained at higher driving frequencies (i.e., 62.5 Hz), compared with lower frequencies, provide better diagnostic performance. In fact, the increase of the frequency of the applied waves results in the increase of effective spatial resolution of MRE [22]. However, high-frequency shear waves are attenuated more rapidly than low-frequency waves. Therefore, scan parameters have to balance between spatial resolution and wave penetration [22].

Ultrasonography has been the first method widely used for the liver stiffness imaging, and its clinical value has been investigated more extensively than that of MRE. Among US techniques, TE seems to present the strongest practical validation. A detailed evaluation of TE limitations was published by Castéra et al., who presented their 5-year experience [5]. In their material failure of liver stiffness, measurement occurred in 3.1 % of all examinations and was independently associated with body mass index (BMI) greater than 30 kg/m^2^, operator experience fewer than 500 examinations, age greater than 52 years, and type 2 diabetes. Unreliable results were obtained in a further 15.8 % of patients and were independently associated with the same factors plus female sex and hypertension [5]. On the other hand, TE was reported to present an excellent interobserver agreement ICC of 0.98 [23].

However, results of stiffness measurement using different US techniques seem to be difficult to compare. In a study by Ferraioli et al., liver point shear wave elastography (PSWE) and TE were compared in healthy volunteers [24]. In the median values, the liver stiffness using PSWE and TE was significantly different (3.5 and 4.4 kPa, respectively). PSWE presented high reproducibility as assessed using concordance correlation coefficient: the intraobserver agreement ranged from 0.83 to 0.96 and the interobserver agreement ranged from 0.83 to 0.93 [24]. However, another study demonstrated that reproducibility of PSWE is dependent on the expertise of the operator and the site of measurement, and that a learning curve using this method should be taken into account [25]. Slightly lower reproducibility was reported for another US technique, real-time elastography, in chronic hepatitis B patients (0.838 for intraobserver reliability and 0.805 for interobserver reliability) [26].

An interesting problem is a direct comparison of MR and US elastography. In a phantom study, stiffness values measured using MRE and TE were highly correlated (*r* ^2^ = 0.93) with the mean difference value of 0.27 kPa and the standard error of 0.58 kPa [27]. An in vivo comparison was carried out by Yoon et al. who examined 94 liver transplantation recipients and 114 liver donors with either MRE or shear wave elastography (SWE) [28]. Considering healthy donors, the mean stiffness values measured with MRE and SWE were significantly different (1.78 and 4.56 kPa, respectively), and the correlation between results collected with both methods was poor (*r* = 0.37). Moreover, MRE and SWE differed significantly regarding within-group coefficients of variation (5.97 and 16.20 %, respectively) [28]. Furthermore, a study with chronic liver disease patients showed that the shear modulus measured by MRE and TE are not equivalent, especially in patients with stiff livers [29].

Our study had some major limitations. Firstly, the number of included subjects was calculated to present variability of stiffness measurement using different methods. Therefore, although the statistical power was sufficient to present differences between segments, our results may not be considered as reference values for Central Europeans. Secondly, we included volunteers whose livers were considered healthy based on medical histories, physical examinations, normal serum ALT levels, and negative result of liver sonography. We believe that histologic proof of the liver condition in volunteers is not justified and that a single site biopsy does not provide sufficient exclusion of the liver pathology to balance the risk of this invasive procedure. However, in our opinion, inclusion of patients with negative clinically indicated liver biopsy would bias the results more, since such subjects usually have some kind of liver pathology that may influence stiffness. It is highly probable that in early subclinical stages of fibrosis differences between methods of stiffness measurement would be similar to our results. Thus, our inclusion criteria seem to sufficient. Finally, placement of ROIs within segments was subjective, and in some cases they might have been located close to the large vessels, which are known to reduce viscoelasticity of the parenchyma.

In conclusion, stiffness of the liver presents a significant variability depending on the region of measurement. The most reproducible method is averaging of several cross sections of the liver. There is a significant variability between stiffness in subjects considered to be healthy, which requires further investigation.
